# Evaluating the impact of data biases on algorithmic fairness and clinical utility of machine learning models for prolonged opioid use prediction

**DOI:** 10.1093/jamiaopen/ooaf115

**Published:** 2025-09-30

**Authors:** Behzad Naderalvojoud, Catherine Curtin, Steven M Asch, Keith Humphreys, Tina Hernandez-Boussard

**Affiliations:** Department of Medicine, Stanford University, Stanford, CA 94305, United States; Department of Surgery, Veterans Affairs Palo Alto Health Care System, Palo Alto, CA 94304, United States; Department of Medicine, Stanford University, Stanford, CA 94305, United States; Center for Innovation to Implementation, Veterans Affairs Palo Alto Health Care System, Palo Alto, CA 94304, United States; Center for Innovation to Implementation, Veterans Affairs Palo Alto Health Care System, Palo Alto, CA 94304, United States; Department of Psychiatry and Behavioral Sciences, Stanford University, Stanford, CA 94305, United States; Department of Medicine, Stanford University, Stanford, CA 94305, United States

**Keywords:** machine learning, algorithmic fairness, data bias, clinical utility, generalizability

## Abstract

**Objectives:**

The growing use of machine learning (ML) in healthcare raises concerns about how data biases affect real-world model performance. While existing frameworks evaluate algorithmic fairness, they often overlook the impact of bias on generalizability and clinical utility, which are critical for safe deployment. Building on prior methods, this study extends bias analysis to include clinical utility, addressing a key gap between fairness evaluation and decision-making.

**Materials and Methods:**

We applied a 3-phase evaluation to a previously developed model predicting prolonged opioid use (POU), validated on Veterans Health Administration (VHA) data. The analysis included internal and external validation, model retraining on VHA data, and subgroup evaluation across demographic, vulnerable, risk, and comorbidity groups. We assessed performance using area under the receiver operating characteristic curve (AUROC), calibration, and decision curve analysis, incorporating standardized net-benefits to evaluate clinical utility alongside fairness and generalizability.

**Results:**

The internal cohort (*N* = 41 929) had a 14.7% POU prevalence, compared to 34.3% in the external VHA cohort (*N* = 397 150). The model’s AUROC decreased from 0.74 in the internal test cohort to 0.70 in the full external cohort. Subgroup-level performance averaged 0.69 (SD = 0.01), showing minimal deviation from the external cohort overall. Retraining on VHA data improved AUROCs to 0.82. Clinical utility analysis showed systematic shifts in net-benefit across threshold probabilities.

**Discussion:**

While the POU model showed generalizability and fairness internally, external validation and retraining revealed performance and utility shifts across subgroups.

**Conclusion:**

Population-specific biases affect clinical utility—an often-overlooked dimension in fairness evaluation—a key need to ensure equitable benefits across diverse patient groups.

## Introduction

The growing use of machine learning (ML) in healthcare has raised important concerns about bias and fairness in patient-level prediction (PLP) models.^1–3^ These biases often originate from imbalanced training data, such as underrepresentation of certain groups, which can lead to inequitable healthcare outcomes and access to resources.^4^^,^^5^ In addition to data issues, the design of algorithms,^6^^,^^7^ and even debiasing strategies themselves^8–10^ can unintentionally reinforce patterns that disadvantage vulnerable subgroups. To ensure fair and clinically meaningful models, both the data and the learning process must be carefully evaluated.

In ML, bias and fairness are closely related but conceptually distinct.^11^ Bias refers to systematic errors in data representation or model development that disproportionately affect certain patient groups. On the contrary, fairness focuses on how equitably a model performs across these groups. Fairness is often measured as performance parity, such as area under the receiver operating characteristic curve (AUROC), area under the precision-recall curve (AUPRC), or Brier scores across groups.^2^ More formal metrics like equalized odds and equal opportunity aim to ensure equitable error rates or true positive rates.^12^^,^^13^ However, these metrics depend on a single decision threshold, which may not reflect how the model performs across the full range of clinical decision points. In practice, such limitations can have tangible consequences—for example, a model that appears fair under metrics like equalized odds at 1 threshold may still under-triage frail patients or over-prescribe opioids for vulnerable groups when applied across varying clinical decision points. In this study, we propose standardized net benefit (SNB) parity to assess fairness by comparing net benefit over a wide range of decision thresholds. This provides a more complete view of fairness and clinical utility across subgroups, helping to ensure that ML models are deployed safely and equitably in real-world healthcare settings.

Achieving a balance between algorithmic fairness and model accuracy is a critical challenge in healthcare ML.^14^ While existing studies emphasize model robustness and generalizability,^15^ often through external validation,^16–18^ they rarely address fairness beyond basic demographic factors such as race or gender. Moreover, efforts to mitigate bias often highlight a potential trade-off: improving fairness may reduce accuracy, while focusing solely on accuracy risks exacerbating existing health disparities.^19^^,^^20^ However, fairness in clinical contexts must also account for characteristics like frailty, comorbidities, and other markers of patient vulnerability, which are often overlooked. To address this, we extend fairness evaluations beyond traditionally underrepresented demographic groups to include clinically vulnerable populations—those at higher risk of harm due to clinical factors—whether or not they are underrepresented in the data. This approach enables us to assess whether models maintain performance and deliver equitable clinical utility across both underrepresented and high-risk populations.

Building on previous work,^2^ which evaluated fairness using classification parity metrics (AUROC and AUPRC) across demographic groups, we extend bias analysis by integrating clinical utility and condition-specific fairness into our evaluation framework, bridging the gap between fairness assessment and real-world decision-making. We apply this framework to electronic health records from the Veterans Health Administration (VHA), the largest integrated healthcare system in the United States, using a previously developed model for predicting prolonged opioid use (POU).^21^ Focusing on POU—a common and clinically important postoperative outcome—we show how population-specific biases, particularly among vulnerable and risk groups, can impact not only model performance but also clinical utility, a dimension often overlooked in fairness evaluations.

## Methods

This study is a retrospective analysis of observational health data that has received approval from the Institutional Review Board at Stanford University. We built our framework for evaluating prediction models developed by the Observational Health Data Sciences and Informatics (OHDSI) PLP R package,^22^ and validated it through a case study to predict patients at risk of POU.^21^

### Fairness, bias, and generalizability framework

**Figure 1.**
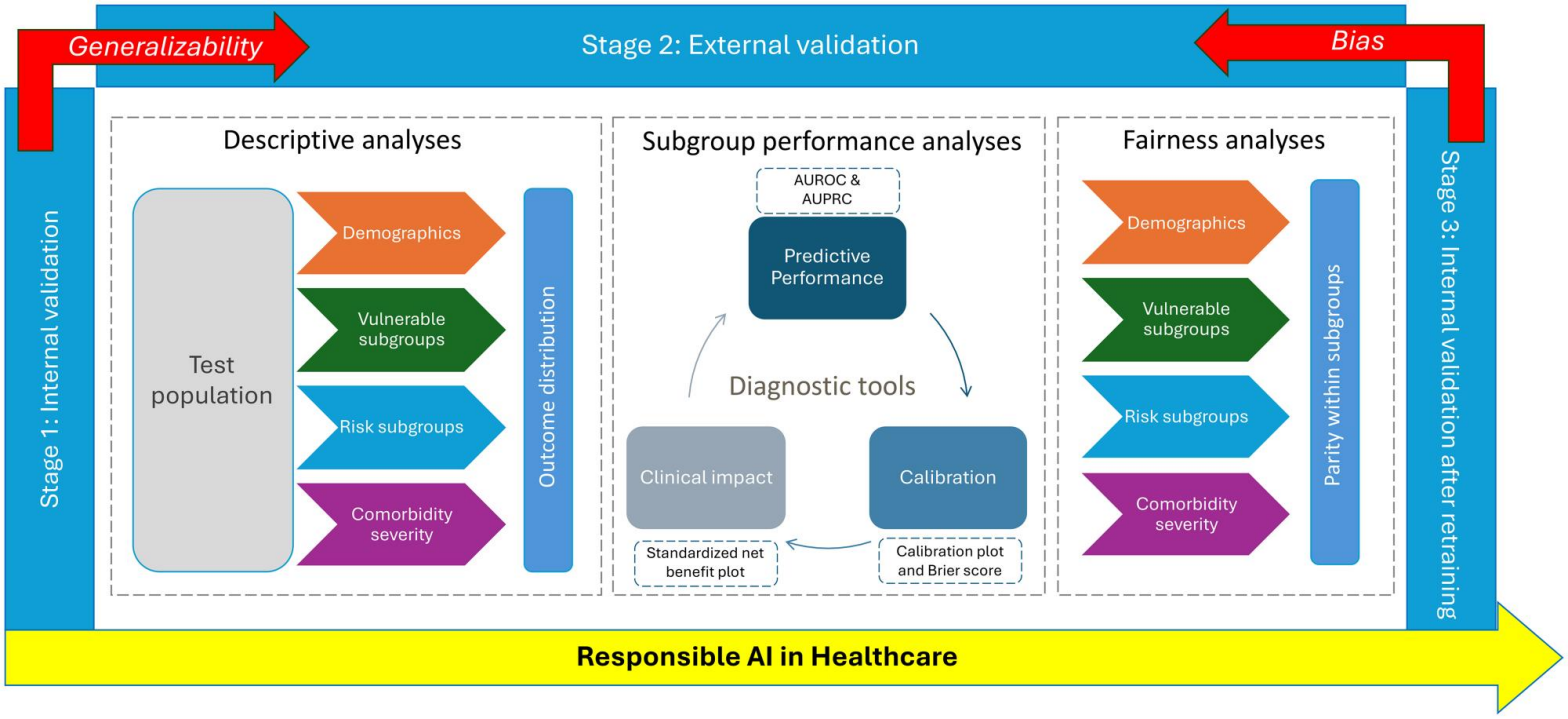
Overall structure of the fairness, bias, and generalizability evaluation framework. The framework comprises three stages: Stage 1 (internal validation), Stage 2 (external validation), and Stage 3 (internal validation after retraining). Comparing Stages 1 and 2 assesses generalizability; comparing Stages 2 and 3 highlights the impact of retraining and potential bias between development and validation datasets. Each stage includes (1) descriptive analyses of the test population across four subgroup domains—demographics, vulnerable subgroups, risk subgroups, and comorbidity severity—along with outcome prevalence distributions; (2) subgroup performance analyses for predictive performance, calibration, and clinical utility (e.g., AUROC/AUPRC, calibration plots and Brier score, and standardized net benefit); and (3) fairness analyses assessing parity within the same subgroup domains.

Figure 1 depicts the overall structure of the proposed evaluation framework. The framework evaluates model generalizability and bias in 3 stages. Stage 1 includes evaluating the internal performance of the model on the dataset used for model training and internal validation, which yields an optimism-corrected estimate,^23^ using bootstrapping with 2000 iterations. Stage 2 applies the model to the external database to evaluate the transportability and generalizability. Stage 3 retrains the model with the external data to reveal any bias compared to Stage 2.

In each stage, subgroup analyses cover 4 categories: demographics (eg, gender, race), vulnerable groups susceptible to harm (eg, diabetes, depression), risk groups with higher chances of outcomes (eg, prior opioid-exposed), and comorbidity severity based on Charlson Comorbidity Index (CCI) (0 = none, 1-2 = mild, 3-4 = moderate, 5+=severe).^24^ Including CCI categories is important to ensure that the models do not discriminate against specific individuals with various conditions or are generalizable across all subgroups. The analysis begins by examining the outcome prevalence distribution across all evaluation subgroups. Model performance is then generated for each subgroup using 3 types of diagnostic tools: model predictive performance, calibration, and clinical utility. Fairness is defined as performance parity among demographic, vulnerable, risk, and comorbidity severity subgroups. However, the performance or calibration parity alone may not fully capture the clinical utility of the model. To address this issue, we incorporated the decision curve analysis using SNB to evaluate the clinical utility of the model across subgroups. This approach helps assess whether observed disparities translate into differences in real-world benefit, thereby supporting more equitable deployment.

### Cohort and data

This study uses observational data from the Stanford Research Repository (STARR) and the nationwide VHA data mapped to the Observational Medical Outcomes Partnership (OMOP) Common Data Model (CDM).^25^^,^^26^ We used the same cohort as the source paper.^21^ We selected adult patients who underwent surgery from 2008 to 2019 and received at least 1 opioid within 30 days before or after surgery. Reserving the first 2 months for standard post-surgical care,^27^ we excluded patients with another surgery within 2-7 months to avoid influencing POU and those who died within a year after surgery. Vulnerable subgroups included diabetes, depression, and obesity. Two low- and high-risk groups were considered for POU: opioid-naive (patients with no opioid prescriptions in the period from 1 year to 30 days before surgery) and opioid-exposed (patients with at least 1 prescription during that period). We used the same 9 opioid ingredients as the source paper to assess exposure and the same outcome definition for POU: at least 1 new opioid prescription between 90 and 180 days after surgery.

### POU risk prediction model

The source paper developed 5 ML models, among which Lasso logistic regression performed the best. We used this model to validate on VHA data with our evaluation framework. The pretrained model used local features from the development database, selected by the chi-square metric. It included demographic and clinical features from 4 domains (condition, procedure, measurement, and drug) over 2 periods: 180 days and 30 days before surgery. We also employed XGBoost with the same features to see how algorithm choice affects fairness. XGBoost uses a different learning algorithm and was the second-best model for predictive performance and calibration in the source paper. Both models were previously trained on 80% of the STARR cohort (41 929 patients) and retrained on 80% of the VHA cohort (397 150 patients).

### Statistical analysis

To assess differences in the distributions of outcome prevalence across the 3 stages, the Kruskal-Wallis test,^28^ was employed, followed by pairwise comparisons using the Wilcoxon test. For binary groups, we utilized the *Z*-score test, and for multi-class groups, the Chi-square test. The predictive performance was measured with AUROC and AUPRC, both with 95% CIs using 2000 bootstrap samples.^29^ Non-overlapping 95% CIs indicated significant subgroup performance differences. We assessed model calibration with the Brier score, reflecting how well predicted probabilities match actual outcomes. Lower Brier scores mean better calibration, with 0 being perfect, while higher scores indicate worse calibration. Along with AUROC and AUPRC, the Brier score helped evaluate model reliability across subgroups.

Decision curve analysis using SNB,^30^ was conducted to assess the clinical utility. We assessed SNB across threshold probabilities ranging from 0% to 80%, reflecting a wide range of plausible clinical decision points and capturing model stability across varying trade-offs between true positives and false positives. This analysis allows us to examine whether the model offers equitable net benefit across subgroups, supporting fairness in real-world decision-making.

## Results

**Figure 2.**
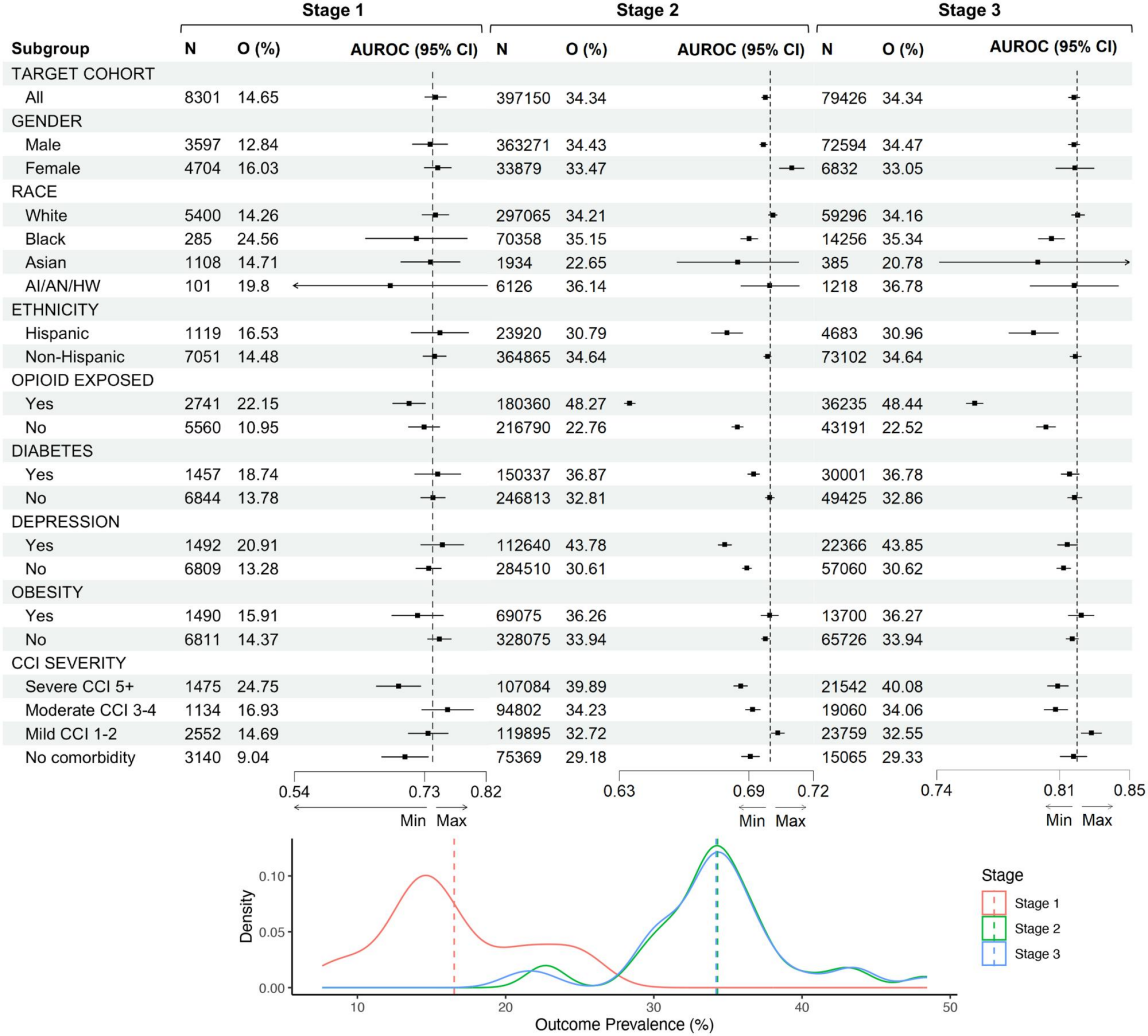
AUROC (area under the ROC curve) for the Lasso model with 95% confidence intervals (CIs) across demographic, vulnerable, risk, and comorbidity severity groups in three stages: (Stage 1) internal validation using 20% of the test set on the STARR dataset; (Stage 2) external validation using 100% of the target cohort on the VHA dataset; and (Stage 3) retraining on the VHA dataset and internally validating using 20% of the VHA test set. The bottom of the figure depicts the distribution of outcome prevalence across three stages. Stage 1 has a significantly different outcome distribution compared to Stages 2 and 3 (*P* < .001). AI/AN/HW stands for American Indian/Alaska Native/Hawaiian.

Figure 2 shows the evaluation framework’s forest plot with 3 stages. Stage 1 evaluates the model’s internal performance on the 20% of the development dataset. Stage 2 assesses generalizability with 100% of external data, showing subgroup performance changes due to data shifts. Stage 3 retrains the model on 80% of external data and tests on 20% to realize model biases. Each stage includes subgroup analyses: demographics, vulnerable, risk, and comorbidity severity groups. From descriptive analysis at each stage, outcome prevalence jumped from 14.65% (*N* = 8301) in Stage 1 to 34.34% (*N* = 397 150) in Stage 2 (*Z*-score test, *P* < .001). Figure 2 also shows that the distribution of outcome prevalence was significantly different between Stages 1 and 2 (Wilcoxon test, *P* < .001), especially in the opioid-exposed risk group.

In Figure 2, AUROC dropped from 0.74 in Stage 1 to 0.70 in Stage 2 (6% reduction, *P* < .001). Predictive performance in Stage 1 was equal across groups, but Stage 2 showed disparities: Hispanic 0.68 vs non-Hispanic 0.70 (*P* < .001), opioid-naive 0.68 vs opioid-exposed 0.63 (*P* < .001), depression 0.68 vs non-depression 0.69 (*P* < .001), and mild comorbidity 0.71 vs severe comorbidity 0.69 (*P* < .001). Average AUROC in Stage 2 was 0.69 (SD = 0.01), slightly below the whole cohort AUROC of 0.70. Despite the drop in AUROC, AUPRCs improved in Stage 2 (average 0.53, SD = 0.05) from Stage 1 (average 0.37, SD = 0.07), with similar group disparities (Figure S1). In Stage 3, retraining the model increased AUROC in the target cohort from 0.70 to 0.82 (0.12 shift). This shift was seen in most of the subgroups, showing improvement from Stage 2 to Stage 3. Stage 3 still had disparities between opioid-naive and opioid-exposed, and mild comorbidity and other comorbidity subgroups.

**Figure 3.**
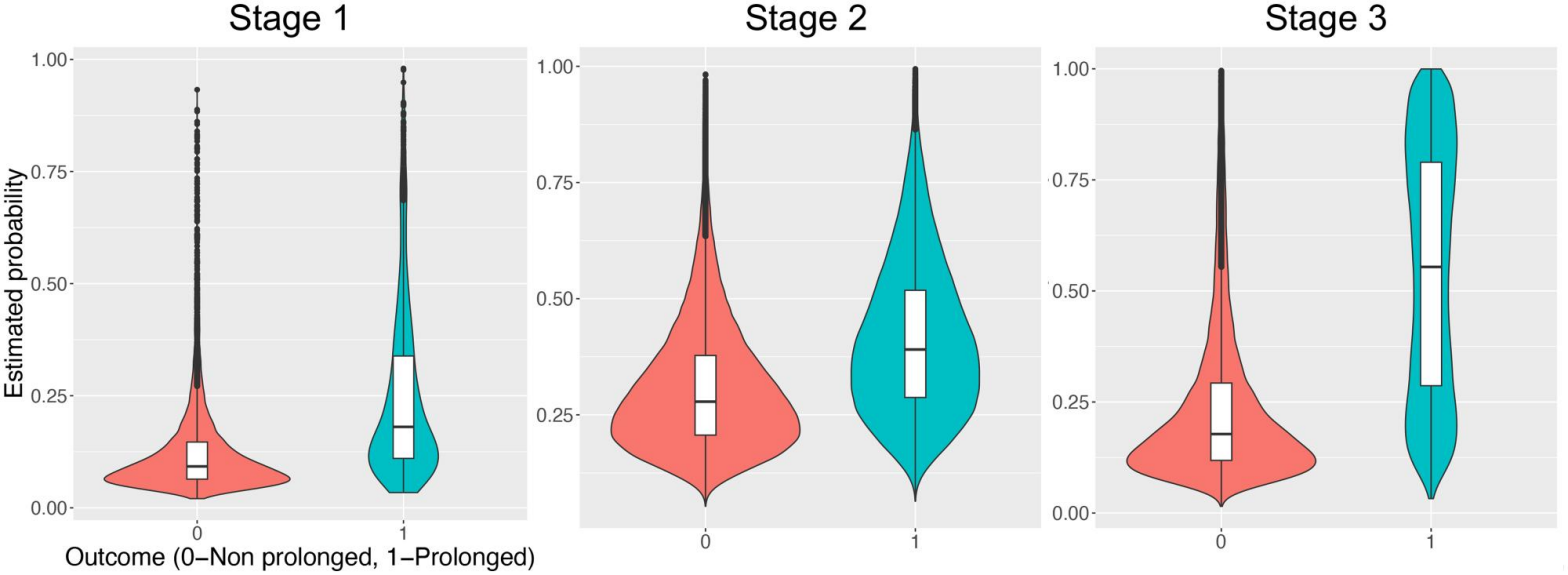
Predicted probability distributions of the Lasso model for prolonged opioid use versus non-prolonged use across three stages: (Stage 1) internal validation using 20% of the test set on the STARR dataset; (Stage 2) external validation using 100% of the target cohort on the VHA dataset; and (Stage 3) retraining on the VHA dataset and internally validating using 20% of the VHA test set.

Figure 3 shows the predicted probability distribution of the POU model across the 3 stages. In both Stages 1 and 2, the predicted probabilities for positive (prolonged) and negative (non-prolonged) classes overlapped substantially, indicating limited class separation. Despite this overlap, the overall trend and shapes of the distributions for each class were similar between Stages 1 and 2, although Stage 2 exhibited a slight shift towards higher predicted probabilities. In Stage 3, class separation improved remarkably, reducing the overlap.

**Figure 4.**
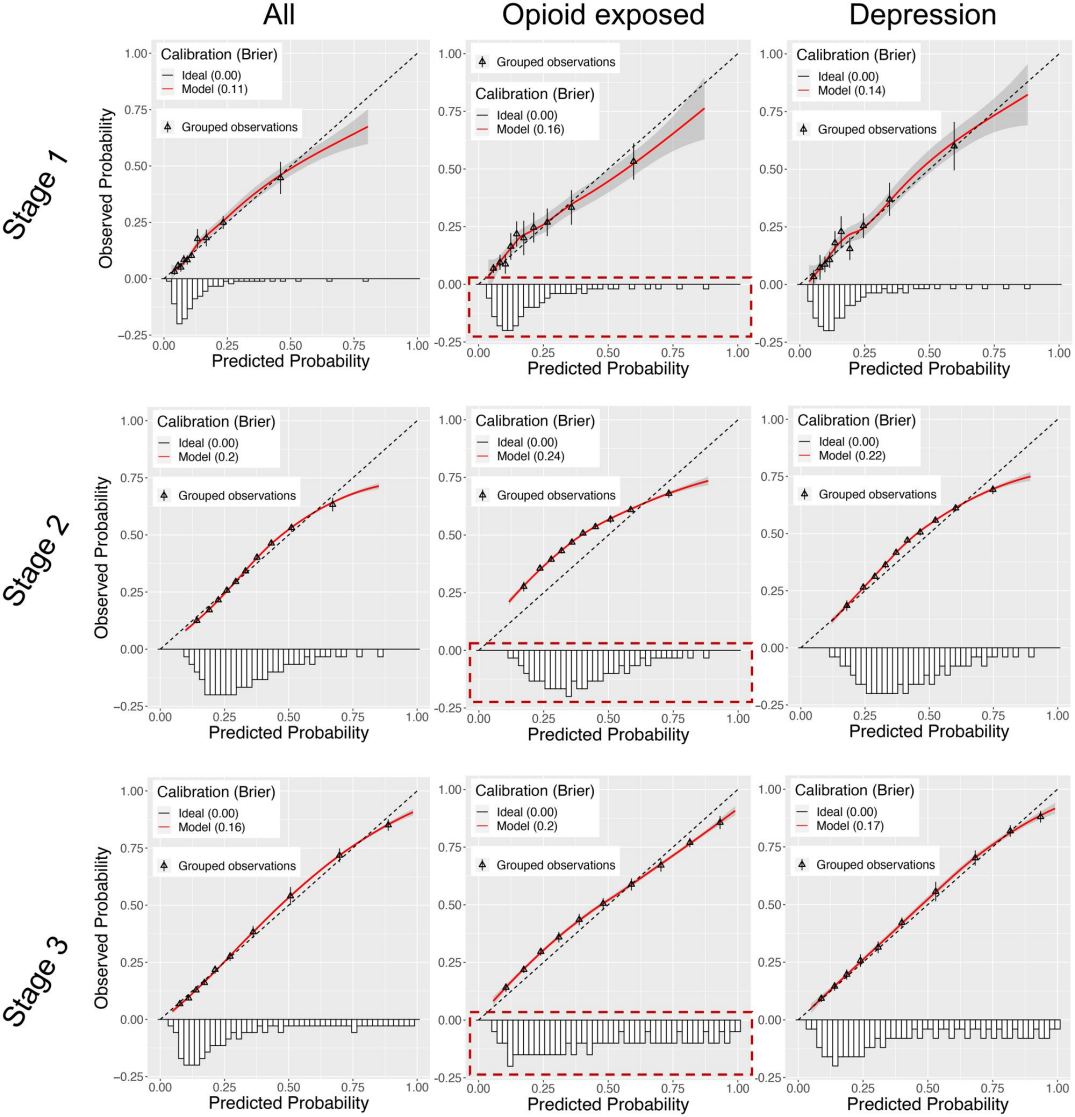
Calibration of the Lasso model for all patients and evaluation subgroups with high prevalence of POU, including opioid-exposed patients and those with depression, across three stages: (Stage 1) internal validation using 20% of the test set on the STARR dataset; (Stage 2) external validation using 100% of the target cohort on the VHA dataset; and (Stage 3) retraining on the VHA dataset and internally validating using 20% of the VHA test set. The dashed box indicates the discrepancy in probability distribution for opioid-exposed patients compared to other evaluation subgroups across the three stages. The Brier scores of the model were reported for each subgroup in parentheses. The 95% confidence intervals for the grouped observations were calculated using the *z*-score and standard error.

Figure 2 showed significant disparities in the opioid-exposed risk subgroup and vulnerable subgroups like diabetes, depression, and severe comorbidity in Stage 2 compared to the overall target cohort. These subgroups had higher POU prevalence rates than in Stage 1. Figure 4 illustrates this impact with calibration plots showing Brier scores and predicted probability distributions for the depression and opioid-exposed subgroups. These 2 subgroups have the highest POU prevalence rates and exhibit the most pronounced miscalibration compared to others. Overall, the model’s predictions are generally well-calibrated across stages but tend to overestimate higher probabilities. This situation varies in the opioid-exposed subgroup, where the model underestimates probabilities below 0.6 in Stage 2. Additionally, there is a clear shift in predicted probability distribution for the opioid-exposed subgroup from lower to higher probabilities between Stages 2 and 3. Calibration plots for demographic variables (gender, ethnicity, and race) and comorbidity groups are shown in Figures S2 and S3, while calibration plots for other clinical subgroups are provided in Figure S10.

**Figure 5.**
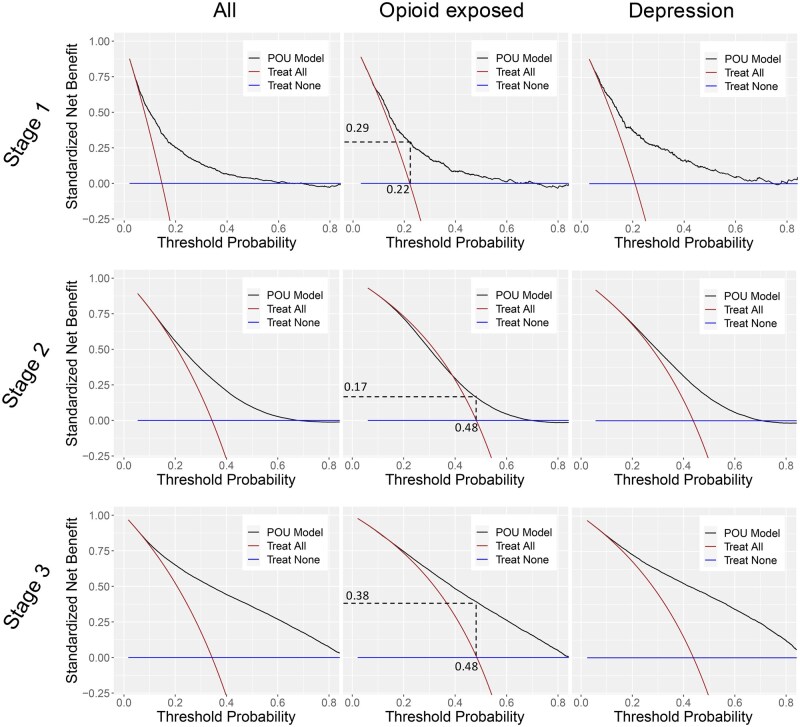
Clinical utility of the Lasso model using standardized net benefit across all patients and evaluation subgroups with high prevalence of POU, including opioid-exposed patients and those with depression, across three stages: (Stage 1) internal validation using 20% of the test set on the STARR dataset; (Stage 2) external validation using 100% of the target cohort on the VHA dataset; and (Stage 3) retraining on the VHA dataset and internally validating using 20% of the VHA test set. The biggest discrepancy was seen in opioid-exposed patients, in whom the clinical utility of the model dropped compared to other evaluation subgroups.

Figure 5 shows decision curve analysis using SNB to evaluate clinical utility/impact across 3 stages for depression and opioid-exposed subgroups with higher POU prevalence, compared to all groups. Compared to the treat-all strategy, the clinical benefits in Stage 1 for all groups were within a threshold range of [0.1-0.5]. In Stage 2, this shifted to [0.2-0.6], and in Stage 3, it widened to [0.1-0.8]. However, the net benefit and threshold ranges were reduced and shifted to higher thresholds in the opioid-exposed subgroup. Where the treat-all strategy had zero net benefit, the threshold increased from ∼0.2 (Stage 1) to ∼0.5 (Stages 2 and 3) and the net benefit in Stage 3 nearly doubled that of Stage 2. Overall, the model provided higher net benefits than “Treat All” and “Treat None” across a smaller range of threshold probabilities in Stage 2 compared to Stage 1, and Stage 3 showed a systematic shift in net benefit across specific thresholds. To assess parity in clinical utility among risk and vulnerable groups, net benefit plots were reported for each group separately. Figures S4-S7 display net benefit plots for opioid-exposed vs opioid-naïve, diabetic vs non-diabetic, depressive vs non-depressive, and obese vs non-obese patients.

To evaluate the impact of algorithms on model fairness, we did fairness analyses using XGBoost (Figures S8 and S9) and compared them with the Lasso model. In Stage 2, AUROC results showed: (1) similar disparities with both algorithms in race, diabetes, and opioid-exposed groups; (2) XGBoost increased disparity in gender and obesity compared to Lasso; and (3) XGBoost reduced disparities in depression and ethnicity.

## Discussion

Building on prior work, this study presents an evaluation framework that expands bias analysis by incorporating clinical utility, showing that biases affect not only fairness and generalizability but also decision thresholds, ultimately limiting the real-world clinical utility of ML models. Using POU prediction as a case study, we demonstrate how outcome prevalence shifts degrade performance and amplify disparities in high-risk groups, such as opioid-exposed patients. Our analysis reveals systematic calibration gaps in external validation on the VHA data: the model tends to underestimate risk for patients with low predicted probabilities and overestimate risk for those with high predicted probabilities. These errors can misinform clinical decisions, emphasizing the importance of subgroup-specific calibration in fairness assessments to ensure equitable clinical utility.

### Addressing fairness in vulnerable and high-risk subpopulations

Our findings show that vulnerable and high-risk subpopulations, specifically severe comorbidity or opioid-exposed groups, experienced significant performance disparities and calibration errors compared to the majority population. These biases persisted even after retraining, despite overall performance improvements (increased AUROC). Notably, the threshold at which the model provided clinical utility was significantly higher for high-risk subgroups. For example, opioid-exposed patients required a threshold probability of ∼.5 to achieve net benefit from the model, compared to ∼.2 in the general cohort. This shift underscores that fairness cannot be achieved through performance optimization alone; explicit subgroup monitoring and intervention—especially for clinically high-risk populations—are essential for equitable deployment, as emphasized in prior work.^31^ One potential solution is to train separate models for opioid-exposed and opioid-naive patients, which may improve both fairness and performance, as supported by previous research.^32^

### Balancing fairness and clinical utility

Balancing fairness and clinical utility requires navigating the trade-offs revealed by our 3-stage evaluation. While retraining improved overall model performance, it did not eliminate disparities for high-risk groups such as opioid-exposed patients. The clinical impact was 2-fold: (1) the threshold for net benefit shifted, making model recommendations less actionable and (2) calibration errors led to systematic underestimation of risk. Together, these findings challenge the assumption that performance gains alone yield equitable clinical utility. Instead, we show that achieving fairness in clinical applications requires both subgroup-specific performance benchmarks and utility-aware optimization—especially in high-stakes settings such as opioid risk stratification. These results further suggest that optimal thresholds may need adjustments across clinical subgroups, potentially achievable through recalibration. Alternatively, fairness-aware methods such as reweighing,^33^ disparate impact removal,^34^ or fair representation learning,^35^^,^^36^ may equalize performance across groups but may obscure group-specific characteristics, affecting both accuracy and utility. While implementing these methods is beyond our current scope, we recognize their potential for addressing fairness gaps while maintaining clinical utility.

### Impact of outcome distribution shifts on model generalizability and fairness

Our 3-stage evaluation framework showed that outcome distribution shifts between development and validation datasets fundamentally altered model behavior, such as generalizability and fairness.^37^ The higher prevalence of POU in the VHA population biased model predictions and contributed to reduced AUROC and increased subgroup disparities during external validation. This shows the model’s performance sensitivity to outcome distribution shifts in real-world settings. High-risk or vulnerable subgroups, such as opioid-exposed and depression patients, experienced greater performance degradation and risk underestimation compared to the general cohort. Notably, AUROC disparities persisted even after retraining, indicating that population shifts do not merely affect overall accuracy but can amplify existing disparities. These findings underscore the importance of evaluating generalizability at the subgroup level, as equitable performance cannot be assumed when applying models across populations with evolving outcome distributions.

### Impact of algorithms on fairness

Our comparison of Lasso and XGBoost shows algorithm-dependent fairness outcomes. While both algorithms showed similar disparity patterns across most groups, XGBoost amplified gender/obesity biases but reduced depression/ethnicity gaps compared to Lasso. This aligns with recent studies showing complex models like XGBoost may learn dataset biases more aggressively than linear models,^38^ yet contradict the assumption that nonlinear models do not inherently worsen all fairness metrics.^39^ Notably, neither algorithm eliminated the core disparity for opioid-exposed patients, reflecting underlying healthcare disparities rather than purely technical limitations, consistent with earlier findings.^1^ These findings suggest that while algorithm choice influences fairness, it cannot fully address systemic biases without incorporating explicit fairness constraints during development.

### Visual tools for understanding model performance and bias

Effective visualization tools are essential for diagnosing model behavior beyond aggregate metrics.^40^^,^^41^ Forest plots revealed subgroup-level disparities in performance that were masked by overall cohort metrics. Calibration plots uncovered systematic miscalibration in high-risk groups, particularly among opioid-exposed patients. Distributions of predicted probabilities illustrated how shifts in data, for example, between training and external validation cohorts, impacted class separability. Finally, decision curve analysis quantified trade-offs in clinical utility across subgroups, highlighting differential benefit for high-risk and vulnerable populations. These visualizations also exposed contradictions—for example, improved AUPRCs in Stage 2 despite AUROC drops—and pinpointed where interventions like retraining in Stage 3 succeeded or failed (eg, persistent opioid-exposed disparities). By linking disparities to specific subgroups and thresholds, these tools transform abstract fairness concerns into actionable insights, underscoring the need for targeted mitigation rather than global adjustments. Ultimately, they enhance prediction validity during external validation and support informed decisions about model deployment.

### Fairness as equitable clinical utility

Our analysis highlights the tension between overall model performance and outcome-based fairness—that is, equitable clinical utility—across subgroups. While Stage 3 retraining improved AUROC, disparities persisted for opioid-exposed, Hispanic, and severe comorbidity subgroups, showing that technical gains did not translate to equitable benefit. Decision curve analysis further quantified these trade-offs: while the model provided net clinical benefit, its utility was unevenly distributed. These findings underscore that fairness cannot be inferred from performance alone; it requires explicit evaluation of who benefits, at what thresholds, in which clinical subgroups. Future work should integrate subgroup-specific thresholds (eg, optimizing net benefit for high-risk groups) to align model outputs with equitable real-world impact.

The results of this study should be considered with its limitations. First, we used an existing model,^21^ inheriting its cohort selection criteria and associated limitations. Second, our bias estimation depends on the external validation population, which might not represent the broader population accurately. Third, our analysis focused on outcome distribution shifts but did not consider covariate distribution shifts. Lastly, differences in population characteristics between the VHA and the development database could affect the model’s performance during external validation and should be noted when interpreting the results. Despite these limitations, the study provides key findings on model validation and generalizability.

## Conclusion

This study highlights the need to evaluate predictive models through a comprehensive lens that incorporates fairness, generalizability, and clinical utility. Population-specific biases and outcome distribution shifts can distort decision thresholds, limit clinical utility, and lead to unequal benefits across patient groups, which is often overlooked in fairness evaluation. Addressing these disparities requires moving beyond traditional performance metrics to include fairness-aware validation, subgroup-specific threshold tuning, and visual tools that clarify model impact. By accounting for both fairness and clinical utility, future models can better support safe and equitable deployment in real-world healthcare settings.

## Supplementary Material

ooaf115_Supplementary_Data

## Data Availability

The datasets used in this study are not publicly available due to ethical restrictions on patients’ personal health data. However, they can be regenerated using the source codes available at the PORPOISE GitHub repository,^42^ for any OMOP CDM database, including the STARR OMOP and VHA OMOP data used in this study.

## Code availability

The source code for this study is available as an R package on GitHub repositories,^43^ making it publicly accessible and usable for evaluating any ML model developed with the OHDSI PLP package.

## References

[1] Obermeyer Z , Powers B, Vogeli C, et al Dissecting racial bias in an algorithm used to manage the health of populations. Science. 2019;366:447-453. 31649194 10.1126/science.aax2342

[2] Röösli E , Bozkurt S, Hernandez-Boussard T. Peeking into a black box, the fairness and generalizability of a MIMIC-III benchmarking model. Sci Data. 2022;9:24. 35075160 10.1038/s41597-021-01110-7 PMC8786878

[3] Chin MH , Afsar-Manesh N, Bierman AS, et al Guiding principles to address the impact of algorithm bias on racial and ethnic disparities in health and health care. JAMA Netw Open. 2023;6:e2345050. 38100101 10.1001/jamanetworkopen.2023.45050 PMC11181958

[4] Bozkurt S , Cahan EM, Seneviratne MG, et al Reporting of demographic data and representativeness in machine learning models using electronic health records. J Am Med Inform Assoc. 2020;27:1878-1884. 32935131 10.1093/jamia/ocaa164 PMC7727384

[5] Cahan EM , Hernandez-Boussard T, Thadaney-Israni S, et al Putting the data before the algorithm in big data addressing personalized healthcare. NPJ Digit Med. 2019;2:78. 31453373 10.1038/s41746-019-0157-2 PMC6700078

[6] Siddique SM , Tipton K, Leas B, et al The impact of health care algorithms on racial and ethnic disparities: a systematic review. Ann Intern Med. 2024;177:484-496. 38467001 10.7326/M23-2960

[7] Paulus JK , Kent DM. Predictably unequal: understanding and addressing concerns that algorithmic clinical prediction may increase health disparities. NPJ Digit Med. 2020;3:99. 32821854 10.1038/s41746-020-0304-9 PMC7393367

[8] FairLearn: An open-source, community-driven project to improve the fairness of AI systems. Accessed July 20, 2025. fairlearn.org

[9] Friedler SA , Scheidegger C, Venkatasubramanian S, et al A comparative study of fairness-enhancing interventions in machine learning. In *Proceedings of the Conference on Fairness, Accountability, and Transparency*. ACM; January 29, 2019:329-338.

[10] Bellamy RK , Dey K, Hind M, et al AI Fairness 360: An extensible toolkit for detecting and mitigating algorithmic bias. *IBM J Res Dev*. 2019;63:1-15.

[11] Mehrabi N , Morstatter F, Saxena N, et al A survey on bias and fairness in machine learning. ACM Comput Surv. 2021;54:1-35.

[12] Hardt M , Price E, Srebro N. Equality of opportunity in supervised learning. Adv Neural Inf Process Syst. 2016;29:3323-3331.

[13] Xu J , Xiao Y, Wang WH, et al Algorithmic fairness in computational medicine. EBioMedicine. 2022;84:104250. 36084616 10.1016/j.ebiom.2022.104250 PMC9463525

[14] Wick M , Panda S, Tristan JB. Unlocking fairness: a trade-off revisited. Adv Neural Inf Process Syst. 2019;32:8783-8792.

[15] de Hond AA , Shah VB, Kant IM, et al Perspectives on validation of clinical predictive algorithms. NPJ Digit Med. 2023;6:86. 37149704 10.1038/s41746-023-00832-9 PMC10163568

[16] Reps JM , Kim C, Williams RD, et al Implementation of the COVID-19 vulnerability index across an international network of health care data sets: collaborative external validation study. JMIR Med Inform. 2021;9:e21547. 33661754 10.2196/21547 PMC8023380

[17] Jin S , Kostka K, Posada JD, et al Prediction of major depressive disorder following beta-blocker therapy in patients with cardiovascular diseases. J Pers Med. 2020;10:288. 33352870 10.3390/jpm10040288 PMC7766565

[18] de Hond AA , Kant IM, Fornasa M, et al Predicting readmission or death after discharge from the ICU: external validation and retraining of a machine learning model. Crit Care Med. 2023;51:291-300. 36524820 10.1097/CCM.0000000000005758 PMC9848213

[19] Dang VN , Cascarano A, Mulder RH, et al Fairness and bias correction in machine learning for depression prediction across four study populations. Sci Rep. 2024;14:7848. 38570587 10.1038/s41598-024-58427-7 PMC10991528

[20] Shanklin R , Samorani M, Harris S, et al Ethical redress of racial inequities in AI: lessons from decoupling machine learning from optimization in medical appointment scheduling. Philos Technol. 2022;35:96. 36284736 10.1007/s13347-022-00590-8 PMC9584259

[21] Naderalvojoud B , Curtin CM, Yanover C, et al Towards global model generalizability: independent cross-site feature evaluation for patient-level risk prediction models using the OHDSI network. J Am Med Inform Assoc. 2024;31:1051-1061. 38412331 10.1093/jamia/ocae028 PMC11031239

[22] Reps JM , Schuemie MJ, Suchard MA, et al Design and implementation of a standardized framework to generate and evaluate patient-level prediction models using observational healthcare data. J Am Med Inform Assoc. 2018;25:969-975. 29718407 10.1093/jamia/ocy032 PMC6077830

[23] Iba K , Shinozaki T, Maruo K, et al Re-evaluation of the comparative effectiveness of bootstrap-based optimism correction methods in the development of multivariable clinical prediction models. BMC Med Res Methodol. 2021;21:9-4. 33413132 10.1186/s12874-020-01201-w PMC7789544

[24] Huang YQ , Gou R, Diao YS, et al Charlson comorbidity index helps predict the risk of mortality for patients with type 2 diabetic nephropathy. J Zhejiang Univ Sci B. 2014;15:58-66. 24390745 10.1631/jzus.B1300109 PMC3891119

[25] Hripcsak G , Duke JD, Shah NH, et al Observational Health Data Sciences and Informatics (OHDSI): opportunities for observational researchers. *Stud Health Technol Inform*. 2015;216:574-578. PMC4815923 26262116

[26] Standardized Data: The OMOP Common Data Model. Accessed July 20, 2025. https://www.ohdsi.org/data-standardization/

[27] Dowell D , Ragan KR, Jones CM, Baldwin GT, Chou R. CDC clinical practice guideline for prescribing opioids for pain—United States, 2022. MMWR Recomm Rep. 2022;71:1-95. 10.15585/mmwr.rr7103a1 PMC9639433 36327391

[28] Vargha A , Delaney HD. The Kruskal-Wallis test and stochastic homogeneity. J Edu Behav Stat. 1998;23:170-192.

[29] Robin X , Turck N, Hainard A, et al pROC: an open-source package for R and S+ to analyze and compare ROC curves. BMC Bioinformatics. 2011;12:77-78. 21414208 10.1186/1471-2105-12-77 PMC3068975

[30] Kerr KF , Brown MD, Zhu K, et al Assessing the clinical impact of risk prediction models with decision curves: guidance for correct interpretation and appropriate use. J Clin Oncol. 2016;34:2534-2540. 27247223 10.1200/JCO.2015.65.5654 PMC4962736

[31] Liu M , Ning Y, Teixayavong S, et al A translational perspective towards clinical AI fairness. NPJ Digit Med. 2023;6:172. 37709945 10.1038/s41746-023-00918-4 PMC10502051

[32] Dwork C , Immorlica N, Kalai AT, et al Decoupled classifiers for group-fair and efficient machine learning. In *Conference on Fairness, Accountability and Transparency*. PMLR; January 21, 2018:119-133.

[33] Krasanakis E , Spyromitros-Xioufis E, Papadopoulos S, et al Adaptive sensitive reweighting to mitigate bias in fairness-aware classification. In *Proceedings of the 2018 World Wide Web Conference*. ACM; April 23, 2018:853-862.

[34] Feldman M , Friedler SA, Moeller J, et al Certifying and removing disparate impact. In *Proceedings of the 21th ACM SIGKDD International Conference on Knowledge Discovery and Data Mining*. ACM; August 10, 2015:259-268.

[35] Zemel R , Wu Y, Swersky K, et al Learning fair representations. In *International Conference on Machine Learning*. PMLR; May 26, 2013:325-333.

[36] Shui C , Chen Q, Li J, et al Fair representation learning through implicit path alignment. In *International Conference on Machine Learning.* PMLR; June 28, 2022:20156-20175.

[37] Singh H , Mhasawade V, Chunara R. Generalizability challenges of mortality risk prediction models: a retrospective analysis on a multi-center database. PLOS Digit Health. 2022;1:e0000023. 36812510 10.1371/journal.pdig.0000023 PMC9931319

[38] Sanh V , Wolf T, Belinkov Y, et al Learning from others’ mistakes: Avoiding dataset biases without modeling them. arXiv preprint arXiv: 2012.01300. 2020.

[39] Tsai TC , Arik S, Jacobson BH, et al Algorithmic fairness in pandemic forecasting: lessons from COVID-19. NPJ Digit Med. 2022;5:59. 35538215 10.1038/s41746-022-00602-z PMC9090910

[40] Austin PC , Putter H, Giardiello D, et al Graphical calibration curves and the integrated calibration index (ICI) for competing risk models. Diagn Progn Res. 2022;6:2. 35039069 10.1186/s41512-021-00114-6 PMC8762819

[41] Hicks SA , Strümke I, Thambawita V, et al On evaluation metrics for medical applications of artificial intelligence. Sci Rep. 2022;12:5979. 35395867 10.1038/s41598-022-09954-8 PMC8993826

[42] PORPOISE GitHub repository: Development and external validation of ml models for identifying patients at risk of postoperative prolonged opioid use. Accessed July 20, 2025. https://github.com/ohdsi-studies/PORPOISE

[43] FairGenEval GitHub repository. Fairness, bias, and generalizability evaluation of OHDSI-developed ML models using OMOP CDM. Accessed July 20, 2025. https://github.com/su-boussard-lab/FairGenEval

