# Supplementary material for: Evaluating the impact of data biases on algorithmic fairness and clinical utility of machine learning models for prolonged opioid use prediction
Source: JAMIA Open. 2025 Sep 30;8(5):ooaf115. doi: 10.1093/jamiaopen/ooaf115 (PMC12483547; doi:10.1093/jamiaopen/ooaf115)
Supplement: ooaf115_Supplementary_Data [file ooaf115_supplementary_data.docx]

**Supplementary Figure 1.** AUPRCs of the Lasso model with 95% confidence intervals (CIs) for demographic, vulnerable, risk, and comorbidity severity groups in three stages: (Stage 1) internal validation using 20% of the test set on the STARR dataset; (Stage 2) external validation using 100% of the target cohort on the VHA dataset; and (Stage 3) retraining on the VHA dataset and internally validating using 20% of the VHA test set. AI/AN/HW refers to American Indian/Alaska Native/Hawaiian.

**Supplementary Figure 2**. Calibration of the Lasso model across demographic groups, including gender, ethnicity, and race, in three stages: (Stage 1) internal validation using 20% of the test set on the STARR dataset; (Stage 2) external validation using 100% of the target cohort on the VHA dataset; and (Stage 3) retraining on the VHA dataset and internally validating using 20% of the VHA test set. The Brier scores of the model were reported for each subgroup in parentheses.

**Supplementary Figure 3.** Calibration of the Lasso model over comorbidity severity subgroups in three stages: (Stage 1) internal validation using 20% of the test set on the STARR dataset; (Stage 2) external validation using 100% of the target cohort on the VHA dataset; and (Stage 3) retraining on the VHA dataset and internally validating using 20% of the VHA test set. The Brier scores of the model were reported for each subgroup in parentheses. The 95% confidence intervals (CIs) for the grouped observations were calculated using the z-score and standard error.

**Supplementary Figure 4.** Clinical utility of the model using standardized net benefits for opioid-exposed vs. opioid-naïve patients.

**Supplementary Figure 5.** Clinical utility of the model using standardized net benefits for diabetic vs. non-diabetic patients.

**Supplementary Figure 6.** Clinical utility of the model using standardized net benefits for depressive vs. non-depressive patients.

**Supplementary Figure 7.** Clinical utility of the model using standardized net benefits for obese vs. non-obese patients.

**Supplementary Figure 8.** AUROCs of the XGBoost model with 95% confidence intervals (CIs) for demographic, vulnerable, risk, and comorbidity severity groups in three stages: (Stage 1) internal validation using 20% of the test set on the STARR dataset; (Stage 2) external validation using 100% of the target cohort on the VHA dataset; and (Stage 3) retraining on the VHA dataset and internally validating using 20% of the VHA test set. AI/AN/HW refers to American Indian/Alaska Native/Hawaiian.

**Supplementary Figure 9.** AUPRCs of the XGBoost model with 95% confidence intervals (CIs) for demographic, vulnerable, risk, and comorbidity severity groups in three stages: (Stage 1) internal validation using 20% of the test set on the STARR dataset; (Stage 2) external validation using 100% of the target cohort on the VHA dataset; and (Stage 3) retraining on the VHA dataset and internally validating using 20% of the VHA test set. AI/AN/HW refers to American Indian/Alaska Native/Hawaiian.

**

**Supplementary Figure 10.** Calibration of the Lasso model over all patients and evaluation subgroups in three stages: (Stage 1) internal validation using 20% of the test set on the STARR dataset; (Stage 2) external validation using 100% of the target cohort on the VHA dataset; and (Stage 3) retraining on the VHA dataset and internally validating using 20% of the VHA test set. The dashed red rectangle indicates the discrepancy in probability distribution for opioid-exposed patients compared to other evaluation subgroups across the three stages. The Brier scores of the model were reported for each subgroup in parentheses. The 95% confidence intervals (CIs) for the grouped observations were calculated using the z-score and standard error.
